# Supplementary material for: Low potassium activation of proximal mTOR/AKT signaling is mediated by Kir4.2
Source: Nat Commun. 2024 Jun 17;15:5144. doi: 10.1038/s41467-024-49562-w (PMC11183202; doi:10.1038/s41467-024-49562-w)
Supplement: Supplementary file 1 — Supplementary Information [file 41467_2024_49562_MOESM1_ESM.pdf]

Supplemental Figures

Supplememtal Figure 1

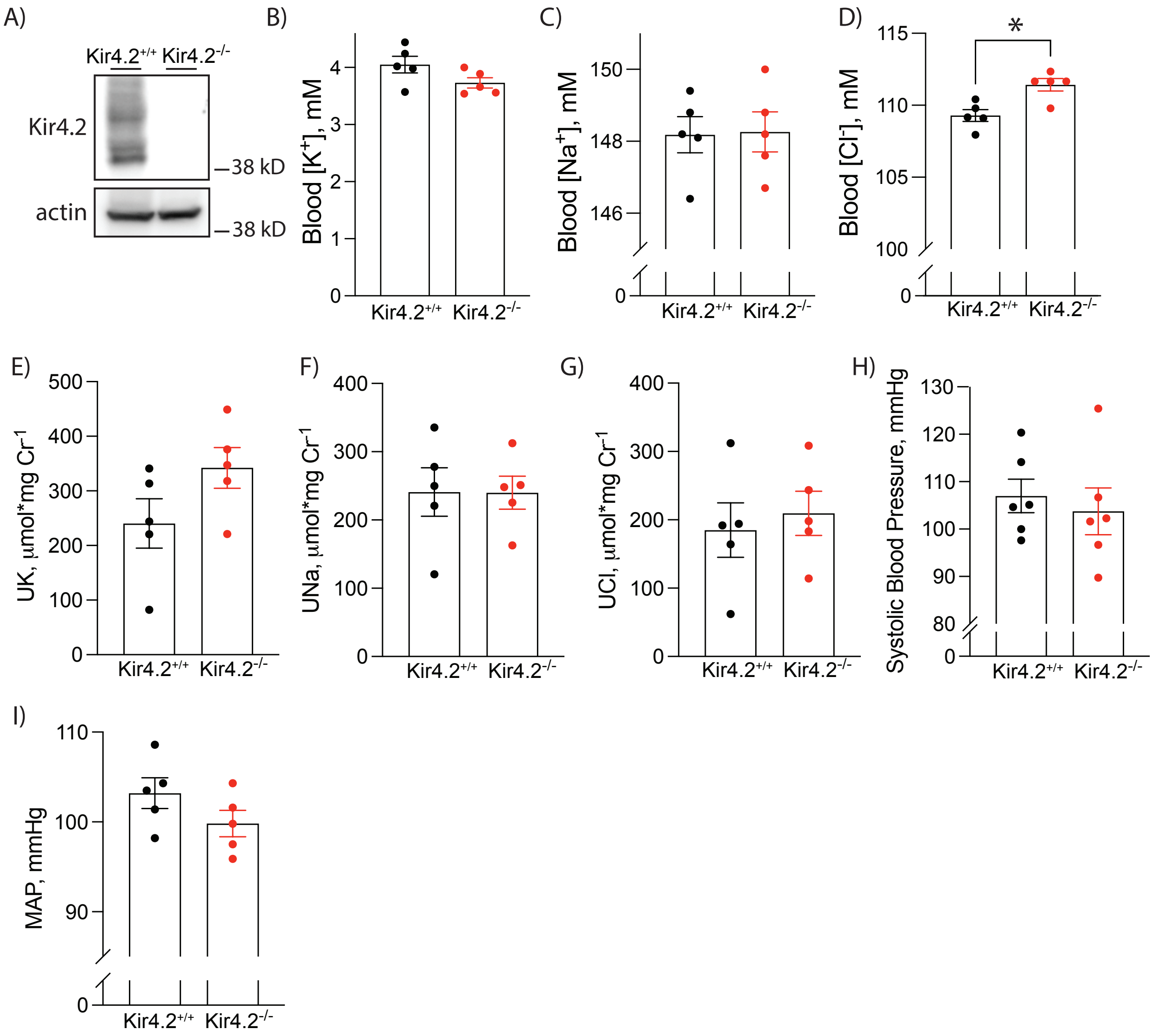

Supplemental Figure 1 Baseline Kir4.2$^{+/+}$ and Kir4.2$^{-/-}$ blood and urine electrolytes. A) Western blot for Kir4.2 protein performed on kidneys from Kir4.2$^{+/+}$ and Kir4.2$^{-/-}$ mice. Blood B) K^+, C) Na^+, and D) Cl^- values (p=0.0069) in Kir4.2$^{+/+}$ and Kir4.2$^{-/-}$ animals on normal diet. Urine E) K^+, F) Na^+, and G) Cl^- values in Kir4.2$^{+/+}$ and Kir4.2$^{-/-}$ animals on normal diet. H) Systolic blood pressure as measured by tail cuff in Kir4.2$^{+/+}$ and Kir4.2$^{-/-}$ animals on normal diet. I) Mean arterial pressure as measured via arterial catheterization in Kir4.2$^{+/+}$ and Kir4.2$^{-/-}$ animals on normal diet. N=5 per group for all except 6 per group in H. *, P<0.05 by unpaired t-test. Data presented as mean +/- sem. All tests are two-sided.

Supplemental Figure 2

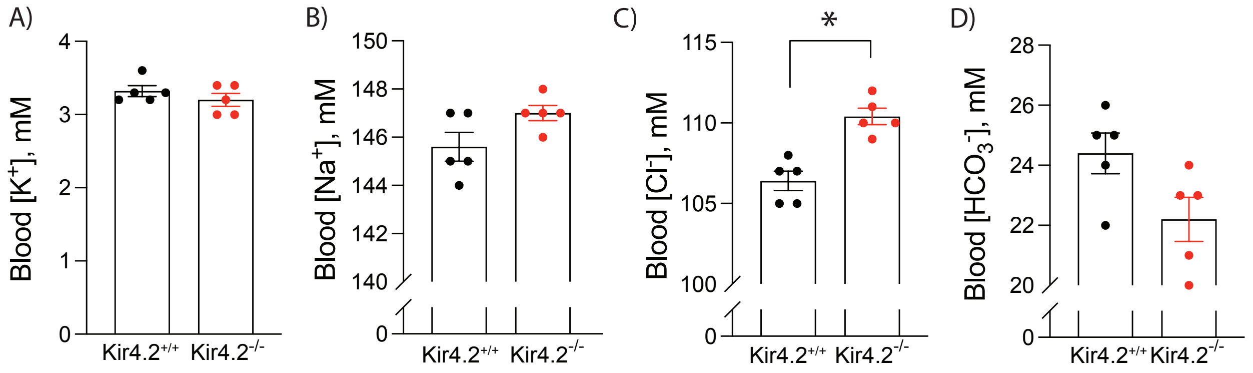

Supplemental Figure 2 Effects of a NaCl-deficient diet on blood electrolytes in Kir4.2$^{+/+}$ and Kir4.2$^{-/-}$ animals. Blood A) K^+, B) Na^+, C) Cl^- (p=0.00095), and D) HCO_3^- in Kir4.2$^{+/+}$ and Kir4.2$^{-/-}$ animals after consuming a NaCl-deficient diet for four days. N=5 per group. Data presented as mean +/- sem. All tests are two-sided.

Supplemental Figure 3

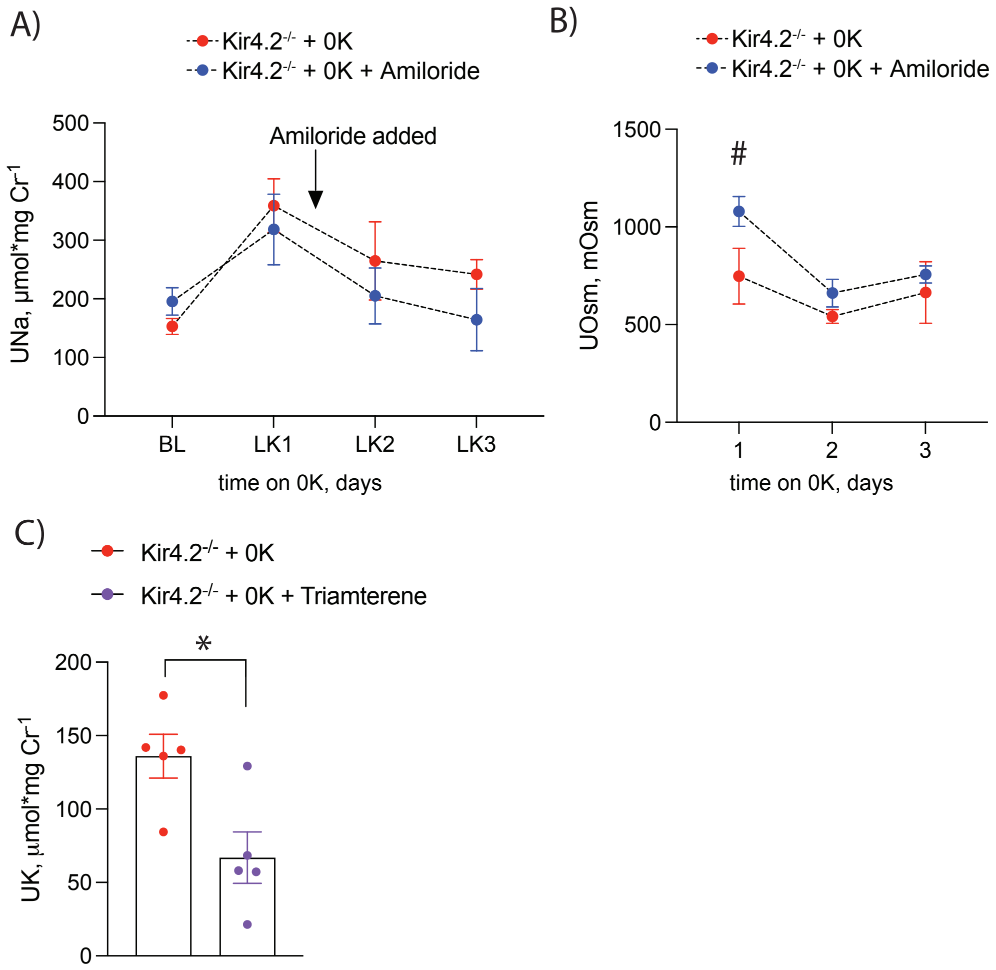

Supplemental Figure 3 Effects of amiloride and triamterene on urine parameter. A) Na^+ excretion at baseline and on 0K diet in Kir4.2$^{-/-}$ mice. Amiloride was added to drinking water at indicated time point. B) Urine osmolality in Kir4.2$^{-/-}$ animals with or without amiloride supplementation at indicated timepoints of 0K diet consumption ($p=0.029$). C) Urine K^+ excretion in Kir4.2$^{-/-}$ animals with or without triamterene treatment on day 3 of 0K feeding ($p=0.017$). N=5 per group for all. * indicates $P<0.05$ by unpaired Student's t-test. # indicates $P<0.05$ by two-way ANOVA with repeated measures followed by Sidak's post-hoc test. Data presented as mean +/- sem. All tests are two-sided.

Supplemental Figure 4

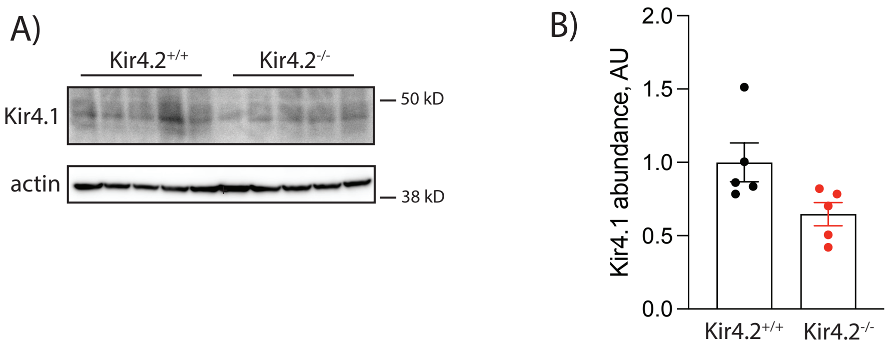

Supplemental Figure 4 A) Western blot for Kir4.1 in Kir4.2$^{+/+}$ and Kir4.1$^{-/-}$ animals on normal diet along with B) quantification. Data presented as mean +/- sem.

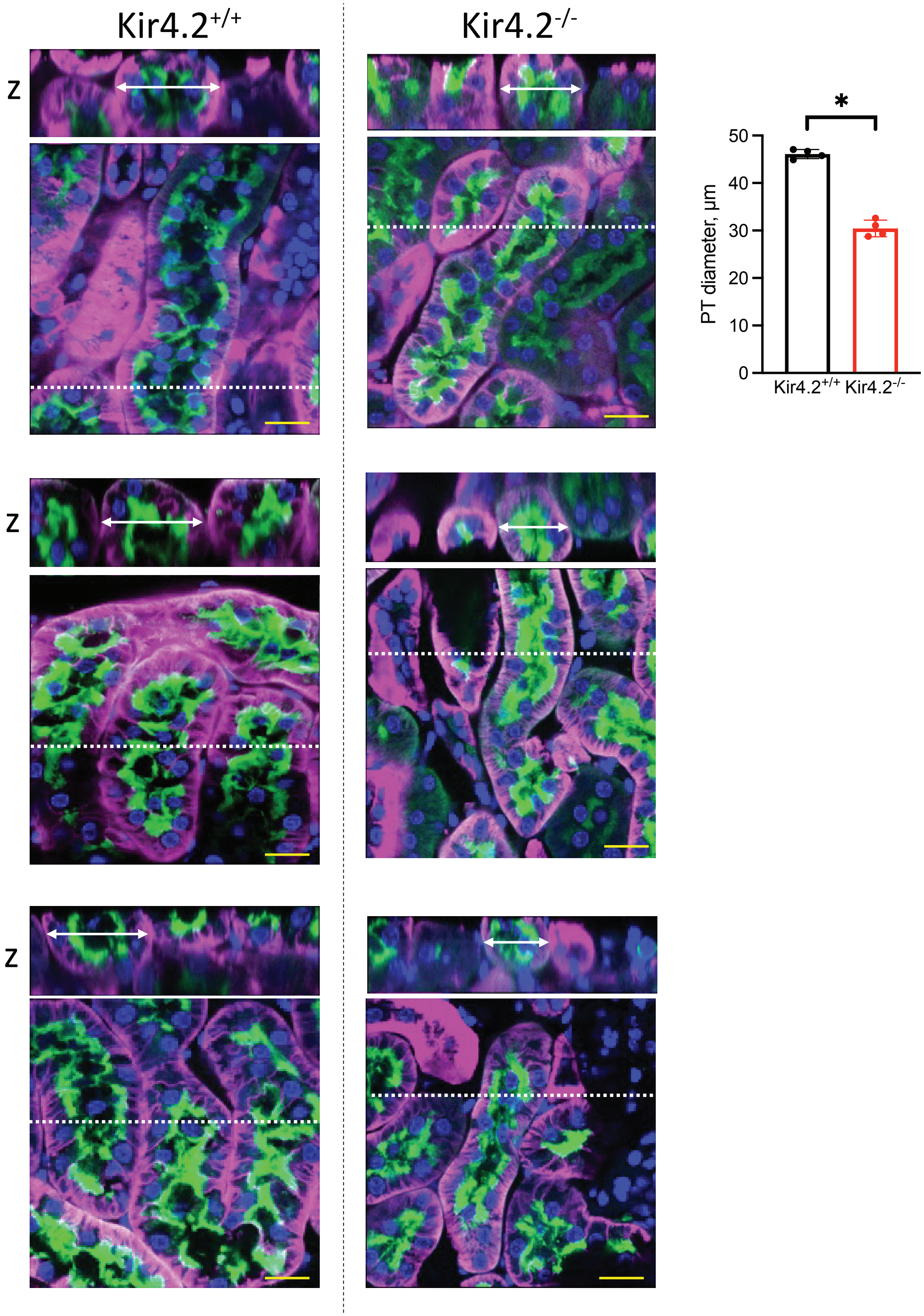

Kir4.2+/+
Kir4.2-/-
Z
Z
Z
*
PT diameter, µm
50
40
30
20
10
0
Kir4.2+/+ Kir4.2-/-

Supplemental Figure 5 Quantification of PT diameter in Kir4.2$^{+/+}$ and Kir4.2$^{-/-}$ animals on 0K. Representative images from control and knockout animals on 0K. LTL was used to localize the PT. Dotted white lines represent level at which the diameter in z-plane was determined. Each dot in bar chart represents the average of 10 tubules measure for a single animal. * indicated $P<0.0001$ by unpaired Student's t-test. Images were acquired using a Super Resolution Airy Scan 2 Detector. N=4 per group. Scale bars = 20μm. Data presented as mean +/- sem. Test is two-sided.

Supplemental Figure 6

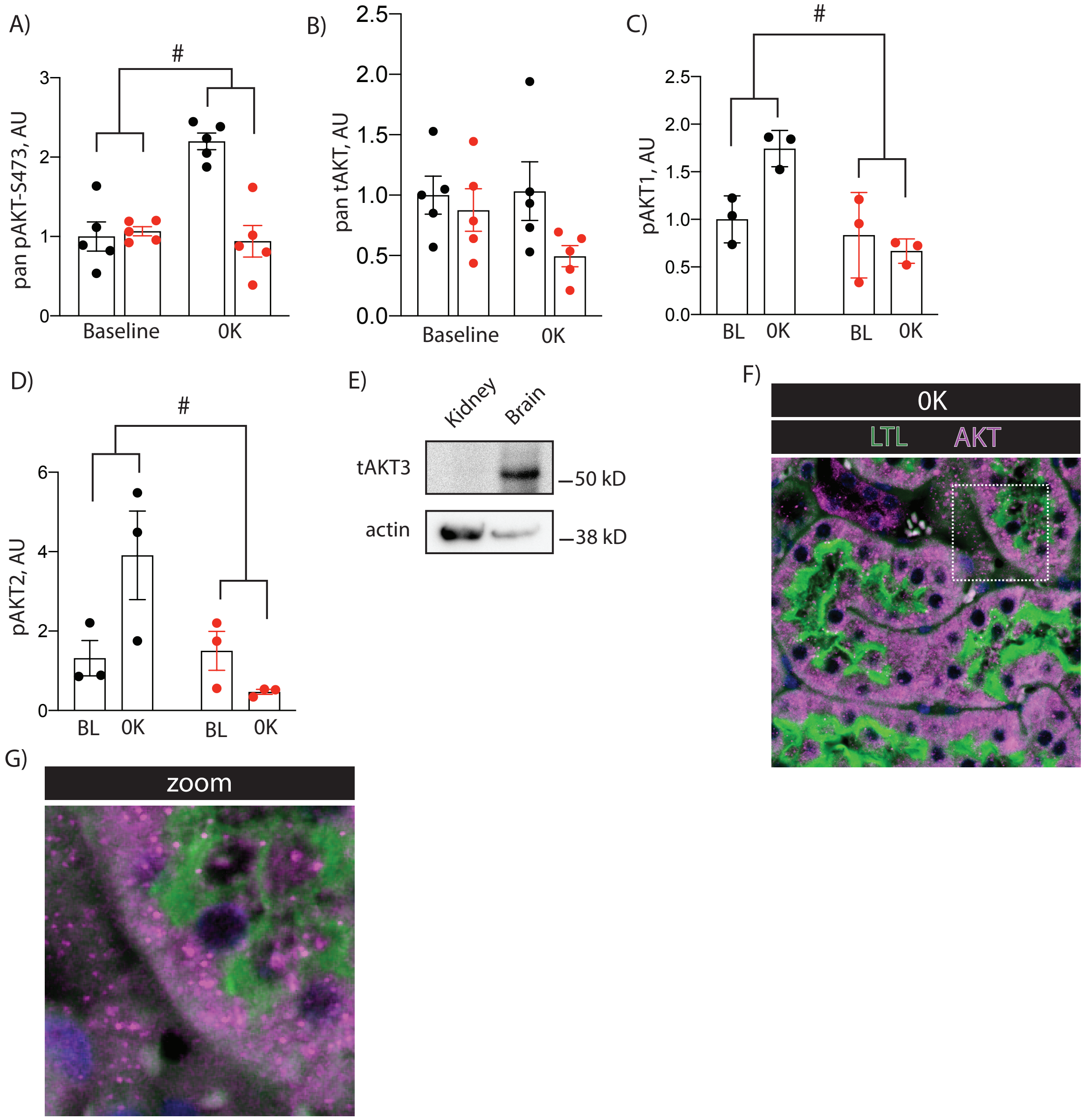

Supplemental Figure 6 Western blot quantification of AKT isoforms and AKT localization. A-D) Western blot quantification for Figure 5a and b (p=0.0004 for interaction in A, p=0.022 for interaction in C, and p=0.023 for interaction in D). E) AKT3 is not detectable in kidney lysate, but is detectable in brain lysate. F) Representative immunofluorescence imaging showing colocalization of AKT and the PT marker LTL from mice on 0K for four days. G) Zoom of section

indicated within white box of F to highlight punctate AKT staining. N=5 per group for A and B. N=3 per group for C and D. N=5 for F. # indicates $P<0.05$ for interaction by two-way ANOVA. Data presented as mean +/- sem. All tests are two-sided.

Supplemental Figure 7

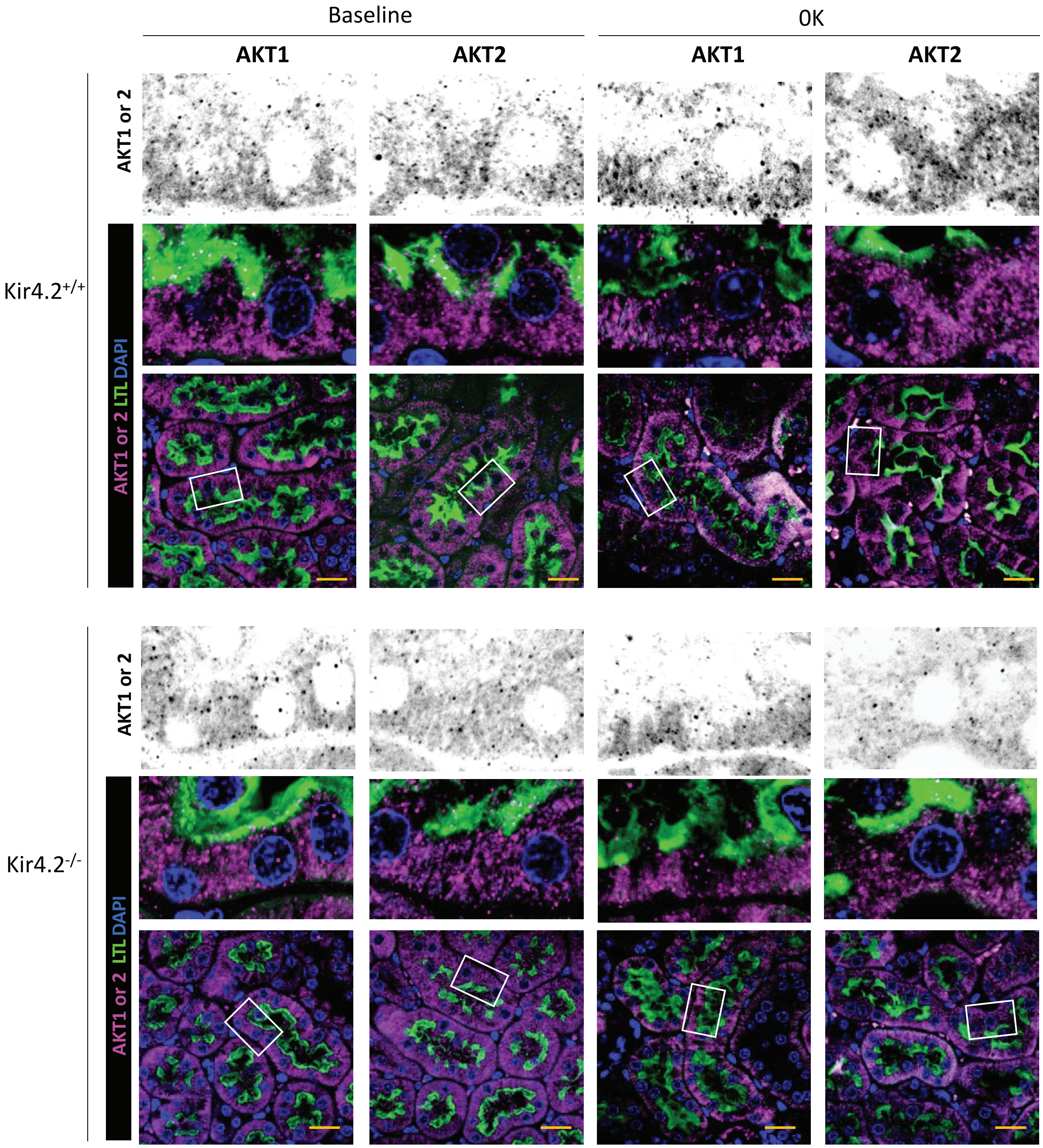

Supplemental Figure 7 AKT1 and AKT2 PT localization in Kir4.2$^{+/+}$ and Kir4.2$^{-/-}$ animals. Immunostaining for AKT1 and AKT2 isoforms in the PT of Kir4.2$^{+/+}$ and Kir 4.2$^{-/-}$ animals under baseline and 0K conditions. LTL was used to localize PT sections. White boxes indicate regions of zoomed panels. Images were acquired using a Super Resolution Airy Scan 2 Detector. Scale bars = 20μm.

Supplemental Figure 8

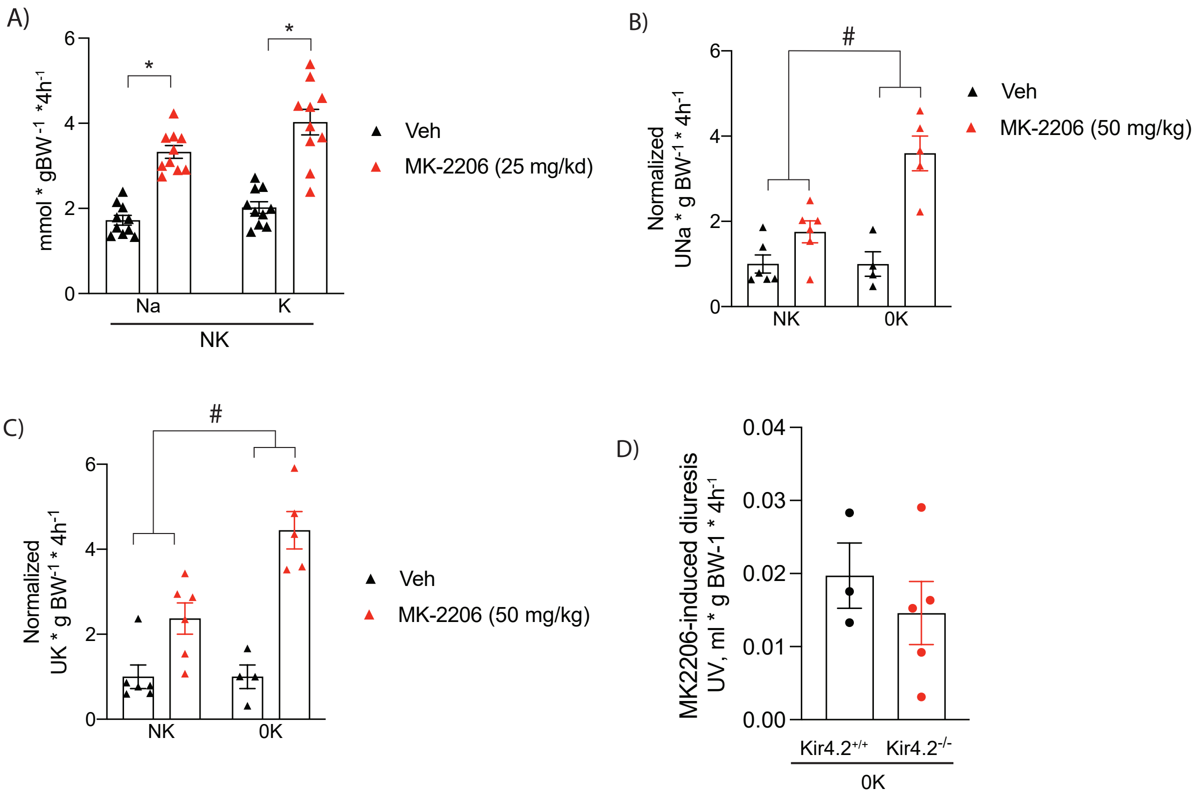

Supplemental Figure 8. Effects of MK-2206 on urine electrolyte excretion. A) Urinary Na^+ and K^+ excretion following treatment with either vehicle (Veh) or MK-2206 at reduced dose of 25 mg/kg in wild-type animals on normal K^+ diet ($p=1x10^{-7}$ and $9x10^{-6}$). B) Urine Na^+ normalized to vehicle-induced excretion from wild-type mice on normal K^+ and 0K diets. Mice were treated with 50 mg/kg MK2206 ($p=0.0063$ for interaction). C) Same as in B, but for urine K^+. Panels B and C are data from Figure 5E and F normalized to the corresponding vehicle-treated group. Vehicle average for each group was set to a value of 1 ($p=0.01$ for interaction). D) MK-2206 diuretic response in Kir4.2$^{+/+}$ and Kir4.2$^{-/-}$ animals. Each data point presented for D is the difference between MK-2206- and vehicle-induced diuresis for each animal. For A, N=10 per group. For B

and C, N= 6, 6, 4, and 5 per group. For D, N=3 for Kir4.2$^{+/+}$ and 5 for Kir4.2$^{-/-}$. * indicated P<0.05 by unpaired Student's t-test. # indicates P<0.05 for interaction by two-way ANOVA. Data presented as mean +/- sem. All tests are two-sided.

Supplemental Figure 9

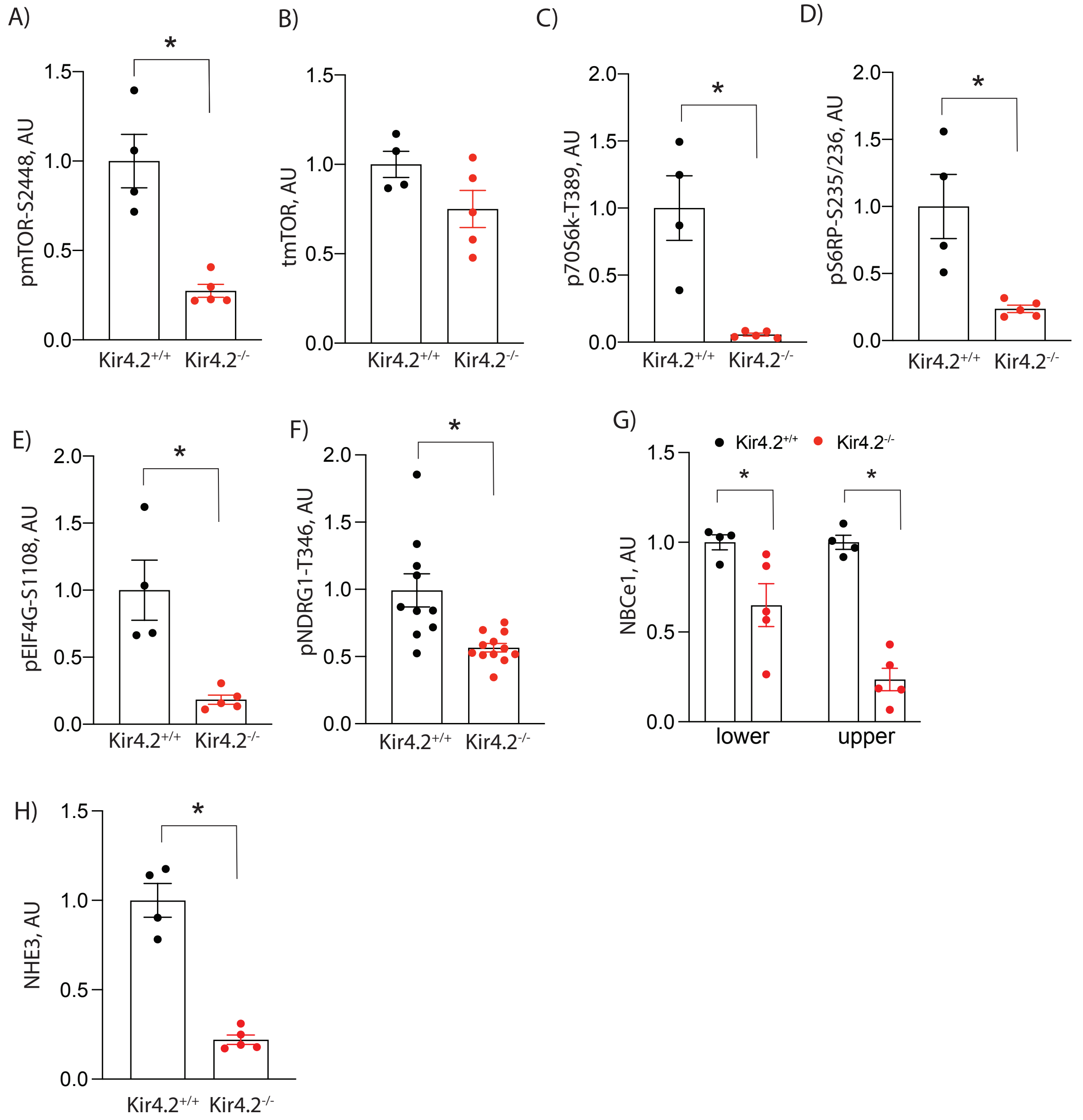

Supplemental Figure 9 Western blot quantification for figure 6b-e. A) Western blot quantification for A) pmTOR (p=0.0012), B) total mTOR, C) p70 S6 kinase (p=0.003), D) pS6 ribosomal protein (p=0.0089), E), pEIF4G (p=0.0048), and F) pNDRG1 (p=0.0016), G) NBCe1 (p=0.041 and $2x10^{-5}$), and H) NHE3 ($p=5x10^{-5}$) in Kir4.2$^{+/+}$ and Kir4.2$^{-/-}$ mice on 0K. * indicates P<0.05 by unpaired Student's t-test. N=4 for Kir4.2$^{+/+}$ and 5 for Kir4.2$^{-/-}$ for all except f where N= 10 and 12. Data presented as mean +/- sem. All tests are two-sided.

Supplemental Figure 10

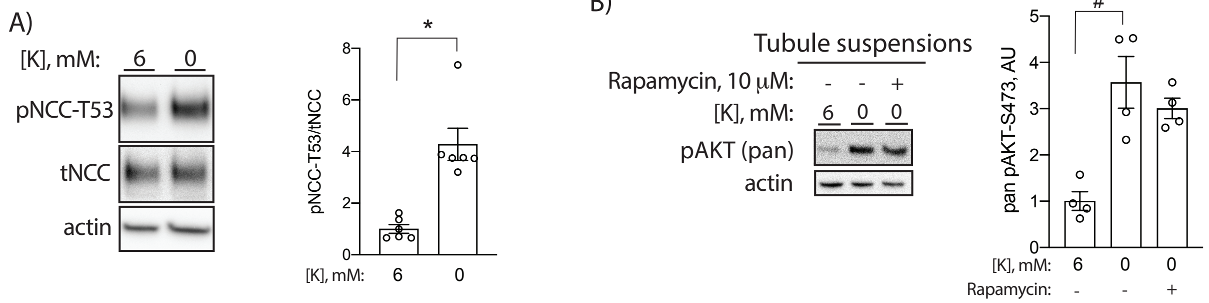

Supplemental Figure 10 Effects of extracellular K^+ reductions on isolated tubule suspensions ex vivo. A) Representative Western blots for NCC from isolated tubule suspensions cultured for 30 min in indicated K^+ concentrations (p=0.0005). B) Representative Western blots from isolated tubule suspensions cultured for 30 min in indicated K^+ concentrations with or without rapamycin (p=0.0014). N=6 per group in A and 4 per group in B. * indicates P<0.05 by unpaired Student's t-test. # indicates P<0.05 by one-way ANOVA with Tukey's post-hoc test. Data presented as mean +/- sem. All tests are two-sided.
