# Supplementary material for: Low potassium activation of proximal mTOR/AKT signaling is mediated by Kir4.2
Source: Nat Commun. 2024 Jun 17;15:5144. doi: 10.1038/s41467-024-49562-w (PMC11183202; doi:10.1038/s41467-024-49562-w)
Supplement: Supplementary file 3 — Reporting Summary [file 41467_2024_49562_MOESM3_ESM.pdf]

nature portfolio

Corresponding author(s): Andrew Terker

Last updated by author(s): May 21, 2024

Reporting Summary

Nature Portfolio wishes to improve the reproducibility of the work that we publish. This form provides structure for consistency and transparency in reporting. For further information on Nature Portfolio policies, see our Editorial Policies and the Editorial Policy Checklist.

Statistics

For all statistical analyses, confirm that the following items are present in the figure legend, table legend, main text, or Methods section.

n/a	Confirmed	
☐	☒	The exact sample size (*n*) for each experimental group/condition, given as a discrete number and unit of measurement
☐	☒	A statement on whether measurements were taken from distinct samples or whether the same sample was measured repeatedly
☐	☒	The statistical test(s) used AND whether they are one- or two-sided<br>*Only common tests should be described solely by name; describe more complex techniques in the Methods section.*
☒	☐	A description of all covariates tested
☐	☒	A description of any assumptions or corrections, such as tests of normality and adjustment for multiple comparisons
☐	☒	A full description of the statistical parameters including central tendency (e.g. means) or other basic estimates (e.g. regression coefficient) AND variation (e.g. standard deviation) or associated estimates of uncertainty (e.g. confidence intervals)
☐	☒	For null hypothesis testing, the test statistic (e.g. *F*, *t*, *r*) with confidence intervals, effect sizes, degrees of freedom and *P* value noted<br>*Give P values as exact values whenever suitable.*
☒	☐	For Bayesian analysis, information on the choice of priors and Markov chain Monte Carlo settings
☒	☐	For hierarchical and complex designs, identification of the appropriate level for tests and full reporting of outcomes
☒	☐	Estimates of effect sizes (e.g. Cohen's *d*, Pearson's *r*), indicating how they were calculated

Our web collection on statistics for biologists contains articles on many of the points above.

Software and code

Policy information about availability of computer code

Data collection	NA
Data analysis	NA

For manuscripts utilizing custom algorithms or software that are central to the research but not yet described in published literature, software must be made available to editors and reviewers. We strongly encourage code deposition in a community repository (e.g. GitHub). See the Nature Portfolio guidelines for submitting code & software for further information.

Data

Policy information about availability of data

All manuscripts must include a data availability statement. This statement should provide the following information, where applicable:

- Accession codes, unique identifiers, or web links for publicly available datasets
- A description of any restrictions on data availability
- For clinical datasets or third party data, please ensure that the statement adheres to our policy

Provide your data availability statement here.

Research involving human participants, their data, or biological material

Policy information about studies with human participants or human data. See also policy information about sex, gender (identity/presentation), and sexual orientation and race, ethnicity and racism.

Reporting on sex and gender	NA
Reporting on race, ethnicity, or other socially relevant groupings	NA
Population characteristics	NA
Recruitment	NA
Ethics oversight	NA

Note that full information on the approval of the study protocol must also be provided in the manuscript.

Field-specific reporting

Please select the one below that is the best fit for your research. If you are not sure, read the appropriate sections before making your selection.

☒ Life sciences ☐ Behavioural & social sciences ☐ Ecological, evolutionary & environmental sciences

For a reference copy of the document with all sections, see nature.com/documents/nr-reporting-summary-flat.pdf

Life sciences study design

All studies must disclose on these points even when the disclosure is negative.

Sample size	Sample sizes were determined based on power calculations taking anticipated effect sizes and a power of 80% at an alpha level of 0.05.
Data exclusions	one data point for a potassium measurement was excluded in figure 1 due to gross hemolysis. this is iindicated in the methods section and figure legend.
Replication	all reported data were based on biological replication as indicated in the corresponding figure legends or methods. all attempts at replicating experiments were successful.
Randomization	experimental groups were determined by using littermates as controls
Blinding	researchers were blinded to experimental conditions where possible

Reporting for specific materials, systems and methods

We require information from authors about some types of materials, experimental systems and methods used in many studies. Here, indicate whether each material, system or method listed is relevant to your study. If you are not sure if a list item applies to your research, read the appropriate section before selecting a response.

Materials & experimental systems

n/a	Involved in the study	
☐	☒	Antibodies
☒	☐	Eukaryotic cell lines
☒	☐	Palaeontology and archaeology
☐	☒	Animals and other organisms
☒	☐	Clinical data
☒	☐	Dual use research of concern
☒	☐	Plants

Methods

n/a	Involved in the study	
☒	☐	ChIP-seq
☒	☐	Flow cytometry
☒	☐	MRI-based neuroimaging

Antibodies

Antibodies used	alpha ENaC rabbit 1:1000 Stressmarq SPC-403 WB<br>gamma ENaC rabbit 1:1000 Stressmarq SPC-405D WB

pNCC-T53 sheep 1:1000 (WB) Phosphosolutions p1311-53 WB
total NCC rabbit 1:10,000 D. Ellison (reference 42) WB
total NKCC2 sheep 1:1000 MRC Dundee S838B WB
Kir4.2 rabbit 1:1000 Alomone Labs APC-058 WB
NHE3 rabbit 1:1000 (WB) Stressmarq SPC-400 WB
NBCe1 rabbit 1:1000 Abcam SPC-400 WB
LTL-fluorescein 1:50 Vector FL-1321-2 IF
pan pAKT-S473 rabbit 1:1000 (WB) Cell signaling 4060 WB
total pan AKT rabbit 1:1000;1:50 (IF) Cell signaling 4691 WB, IF
pAKT1-S473 rabbit 1:1000 Cell signaling 9018 WB
total AKT1 rabbit 1:1000; 1:50 (IF) Cell signaling 2938 WB, IF
pAKT2-S474 rabbit 1:1000 Cell signaling 8599 WB
total AKT2 rabbit 1:1000; 1:50 (IF) Cell signaling 3063 WB, IF
total AKT3 rabbit 1:1000 Cell signaling 4059 WB
pmTOR S2448 rabbit 1:1000 Cell signaling 5536 WB
total mTOR rabbit 1:1000 (WB); 1:50 (IF) Cell signaling 2983 WB, IF
pTSC2-T1462 rabbit 1:1000 Cell signaling 3617 WB
pP70 S6 kinase - T389 rabbit 1:1000 Cell signaling
9205 WB
pS6 S235/236 rabbit 1:1000 Cell signaling 2211 WB
pEIF4g S1108 rabbit 1:1000 Cell signaling 2441 WB
pNDRG1 T346 rabbit 1:1000 Cell signaling 5482 WB
Kir4.1 rabbit 1:1000 Alomone Labs APC-035 WB
Na/KATPase rabbit 1:50 Invitrogen ST0533
actin mouse 1:3000 Millipore A1978 WB

Validation	Validation was based on published reports where Ab was tested on knockout animals as cited in our Table 1 or based on observed molecular weight in our data, published reports, and on the supplier's website.

Animals and other research organisms

Policy information about studies involving animals; ARRIVE guidelines recommended for reporting animal research, and Sex and Gender in Research

Laboratory animals	Mice were used for experimentation were aged 8-12 weeks, on a C57Bl/6 background.
Wild animals	NA
Reporting on sex	Mice were used for experimentation were aged 8-12 weeks, on a C57Bl/6 background.
Field-collected samples	NA
Ethics oversight	All animal experiments were performed in accordance with the guidelines and with the approval of the Institutional Animal Care and Use Committee of Vanderbilt University Medical Center.

Note that full information on the approval of the study protocol must also be provided in the manuscript.

Plants

Seed stocks	NA
Novel plant genotypes	NA
Authentication	NA
